# Individual fMRI maps of all phalanges and digit bases of all fingers in human primary somatosensory cortex

**DOI:** 10.3389/fnhum.2014.00658

**Published:** 2014-09-02

**Authors:** Meike A. Schweisfurth, Jens Frahm, Renate Schweizer

**Affiliations:** ^1^Biomedizinische NMR Forschungs GmbH am Max-Planck-Institut für Biophysikalische ChemieGöttingen, Germany; ^2^Cognitive Neuroscience Laboratory, German Primate CenterGöttingen, Germany

**Keywords:** across-digit map, BA 3b, fMRI, human, individual, intra-digit map, complete mapping, somatotopy

## Abstract

This study determined the individual maps of all fingers in Brodmann area 3b of the human primary somatosensory cortex in a single fMRI session by tactile stimulation at 19 sites across all phalanges and digit bases of the 5 right-hand digits. To quantify basic features of the digit maps within and across subjects, we applied standard descriptive measures, but also implemented a novel quantitative analysis. This so-called Direction/Order (DiOr) method tested whether subjects exhibited an ordering of peak fMRI representations along their individual direction of alignment through the set of analyzed phalanges and whether these individual directions were similar across subjects. Across-digit analysis demonstrated that for each set of homologous phalanges, the D5-to-D1 representations were successively represented along a common direction of alignment. Hence, the well-known mediolateral D5-to-D1 somatotopy was not only confirmed for the distal phalanges (p1), but could also be shown for the medial (p2) and proximal phalanges (p3). In contrast, the peak activation for the digit bases (p4) only partly elicited that digit succession. Complementary, intra-digit analysis revealed a divergent picture of map topography for the different digits. Within D5 (and in a trend: D4), an ordered p1-to-p3 succession was found across subjects, pointing to a consistent intra-digit somatotopy for D5, with p3 generally found medial-posterior to p1. In contrast, for D1, D2, and D3, most subjects did not present with ordered p1-to-p3 maps nor were directions of alignment similarly oriented between subjects. These digits therefore exhibited highly diverse representation patterns across subjects.

## Introduction

Primary sensory cortices are characterized by topographic maps which represent the peripheral receptor sheet in a highly ordered fashion (Kaas, 1997). In the auditory cortex, frequency is encoded along a single tonotopic axis, whereas the retinotopic map in the primary visual cortex incorporates two perpendicular visual-field axes to encode eccentricity and polar angle. The somatotopy of the human primary somatosensory cortex (SI), first described by Penfield's somatosensory homunculus (Penfield and Boldrey, 1937; Penfield and Rasmussen, 1950), comprises the tactile body-surface representation along the postcentral gyrus, representing the digits D5 (little finger) to D1 (thumb) in medial-to-lateral succession. Electrophysiological mapping of the hand area in anesthetized primates implies a second axis perpendicular to the first, representing the phalanges within a digit, with the fingertip or distal phalanx (p1) being represented toward the Brodmann area (BA) 3a/3b border and the proximal parts of the digit toward the BA 3b/1 border (for new-world owl monkeys, see Merzenich et al., 1978; for old-world macaques, see Kaas et al., 1979 and Nelson et al., 1980). Electrophysiological mapping data from awake monkeys is sparse, but in awake macaques, the intra-digit representations of the phalanges were found to be less ordered (Iwamura et al., 1983). Neurons with receptive fields for the proximal phalanges were still found close to the BA 3b/1 border, whereas representations of distal phalanges were not limited toward BA 3a but present across the entire respective digit's BA 3b strip.

In humans, several studies confirmed the medial-to-lateral succession of the digit representations for D5 through D1 in SI, with functional magnetic resonance imaging (fMRI) during tactile or electrical stimulation delivered to the distal phalanx (e.g., Nelson and Chen, 2008; Schweizer et al., 2008; Sanchez-Panchuelo et al., 2010). So far, similar across-digit somatotopies of the second or third phalanges have not been investigated in humans by imaging. Attempts to obtain intra-digit maps, by stimulating the phalanges within a digit, have so far mostly been limited to single digits and yielded inconsistent results both with fMRI and MEG (magnetoencephalography). An overview of all previous studies on intra-digit mapping in individual humans is given in Table 1. In an fMRI study, Blankenburg et al. (2003) stimulated the three phalanges (p1 to p3) and the digit base (p4) of D3. Combining the data of eight subjects into a group analysis, they found a succession of p1-to-p4 activations within BA 3b, pointing toward the BA 3b/1 border. This pattern is similar to the intra-digit somatotopy obtained in anesthetized owl monkeys (Merzenich et al., 1978). Focusing on the left hand, Sanchez-Panchuelo et al. (2012) applied the traveling-wave stimulation and analysis approach (originally developed for mapping the visual cortex, Sereno et al., 1994) to the phalanges and base of D2. They showed individual activation patterns in which they visually identified a mirror-reversal pattern for p1 to p3 and p3 to p1 across SI in 4 out of 6 subjects, suggesting an ordering similar as in anesthetized macaques (Nelson et al., 1980). The group further showed similar phase maps in 3 out of 4 subjects jointly for the digits D2 to D4, by simultaneously stimulating the phalanges p1 to p3 (sequentially) of these three digits (Sanchez-Panchuelo et al., 2014). In 2004, Overduin and Servos explored individual intra-digit phase bands for D1, D2, and D4 in BA 3b, but stated that they did not investigate across-subjects somatotopy due to difficulties in map interpretation caused by limitations in cortical surface sampling. Also analyzing individual maps elicited by tactile stimulation of the fingertip (p1) and the base (p4) of the little finger (D5) and the index finger (D2), our group could show that in BA 3b the base presentation was consistently located medial to the fingertip representation for D5, whereas for D2 no consistent across-subject pattern was observed between the representations of the fingertip and the base (Schweisfurth et al., 2011).

**Table 1:** **Overview of fMRI and MEG studies of intra-digit topology on individual human subjects**.

**Publication**	**Technique**	Subjects^*^	**Hand**	**Digits**	**Phalanges**	**Cortical region**	**Reported intra-digit pattern**
Blankenburg et al., 2003	fMRI	6	Right (dominant)	D3	p1 to p3^**^	BA 3b and BA 1	Ordered p1 to p3 representations orthogonal to central sulcus (inferior to superior, statistically significant)
Overduin and Servos, 2004	fMRI	6	Right (dominant)	D1	p1 to p3	BA 3a, BA 3b, and BA 1	Only individual results, across-subject somatotopy not evaluated
				D2			
				D4			
Schweisfurth et al., 2011	fMRI	10	Right (dominant)	D2	p1 and p4	BA 3b	No consistent topology between p1 and p4 representations across subjects
				D5			Representation of p4 medial to p1 (statistically significant)
Sanchez-Panchuelo et al., 2012	fMRI	6	Left	D2	p1 to p4	BA 3b, 1, 2	Ordered p1 to p3 representations orthogonal to central sulcus, with reversals between BAs, in 4 out of 6 subjects
Sanchez-Panchuelo et al., 2014	fMRI	4	Left	D2 to D4 simultaneously	p1 to p4	BA 3b, 1, 2	Ordered p1 to p3 representations orthogonal to central sulcus, with reversals between BAs, in 3 out of 4 subjects
Current study	fMRI	11	Right (dominant)	D1	p1 to p3	BA 3b	No consistent topology between p1, p2, and p3 representations across subjects
		14		D2			No consistent topology between p1, p2, and p3 representations across subjects
		12		D3			No consistent topology between p1, p2, and p3 representations across subjects
		11		D4			Ordered p1–p3 representations along course of CS (anterior to posterior, statistical trend)
		11		D5			Ordered p1–p3 representations along course of CS (lateral-anterior to medial-posterior, statistically significant)
Hashimoto et al., 1999a	MEG	11	Right	D2	p1 to p3 (plus three points at palm)	BA 3b	No consistent topology between equivalent current dipole (ECD) p1, p2, and p3 representations across subjects
Hashimoto et al., 1999b	MEG	14	Right	D2	p1 to p3 (plus two points at palm)	BA 3b	No consistent topology between equivalent current dipole (ECD) p1, p2, and p3 representations across subjects
Hlushchuk et al., 2004	MEG	11	Right (dominant)	D2	p1 and p3	BA 3b	Representation of p3 superior to p1 (statistically significant)
Tanosaki and Hashimoto, 2004	MEG	17	Right	D3	p1 and p3	BA 3b	Representation of p3 lateral to p1 (statistically significant)

* *Only subjects who showed significant activation in respective area*.

** *Stimulation involved p1 to p4 and palm. Because only one subject showed significant activation for all five locations, only p1 to p3 were taken into account for this table*.

Using MEG and stimulating p1 to p4 of the index finger, Hashimoto et al. (1999a,b) repeatedly observed non-ordered phalanx representations, whereas Hlushchuk et al. (2004) found p3 to be represented superior to p1, in qualitative agreement with the monkey data. Exploring the phalanx representations of the middle finger, Tanosaki and Hashimoto (2004) localized the p3 representation lateral to that of p1, in contrast to the Blankenburg et al. (2003) where p3 was found superior to p1.

To gain further insight into the individual intra-digit maps of specific digits, we used fMRI to determine the BA 3b representations for all phalanges of all five digits of the right, dominant hand at an isotropic spatial resolution of (1.5 mm)^3^. Optimization of the stimulation paradigm enabled us to measure all phalanges and the digit base of each finger in one fMRI measurement and the data from all 19 stimulation sites within one fMRI session. Based on these data the general layout as well as the individual variations of the BA 3b maps of the entire finger area could be investigated across a larger cohort of healthy subjects.

The large number of stimulation sites also called for a more quantitative approach in describing the spatial pattern of phalanx representations than just visual inspection or manual delineation. With the Euclidean distance measure we specifically analyzed the spatial locations of the peak activations in each subject along two axes: First, along the 5 digits (across-digit map), where the distances between the representations of the homologous phalanges (distal phalanges, second phalanges, third phalanges, digit base) were explored separately across digits, and secondly, within individual digits (intra-digit map), considering the distances between the different phalanges (p1 to p4).

To quantify the main directions along which the representations were oriented and to compare these individual BOLD activation patterns across subjects, we used a novel analysis approach termed Direction/Order (DiOr) method. Principle component analyses were applied to the peak-activation locations for either the across-digit maps or the intra-digit maps. Hereby, we could identify the main direction along which the homologous phalanges across digits or the different phalanges within a digit were distributed in each subject and compare these individual directions statistically across subjects. In addition to this main axis along which the phalanx presentations were distributed, we also assessed whether activations associated with neighboring skin positions were ordered along the subject's main direction. If the main directions were similar across subjects and individual patterns were generally ordered along the subjects' main directions, we assumed consistent across-subject somatotopy for the respective phalanx or digit.

## Materials and methods

### MRI

Eighteen healthy subjects (6 women, range 21–30 years, mean 27 ± 2.5 years) participated in two MRI sessions. The study was approved by the ethics committee of the Georg-Elias-Müller-Institute of Psychology, Göttingen University, and informed written consent was obtained before each session. All subjects were right-handed according to the Edinburgh Inventory (laterality index 0.9 ± 0.1, Oldfield, 1971). Magnetic resonance imaging (MRI) was performed at 3 T (TIM Trio, Siemens Healthcare, Erlangen, Germany) using a 32-channel head coil. The first session incorporated a whole-brain structural MRI measurement, a partial-volume localizer fMRI measurement for delineating the presumed digit-representation area along the central sulcus, whole-brain echo-planar imaging (EPI), and MR angiography. The second session consisted of 5 fMRI measurements, each mapping the phalanges within a single digit.

Structural MRI was based on sagittal T1-weighted 3D MPRAGE (magnetization-prepared rapid gradient-echo) acquisition [repetition time (TR) = 2530 ms, echo time (TE) = 3.4 ms, flip angle = 7°, acquisition matrix = 256 × 256, 160–192 partitions, resolution = 1 × 1 × 1 mm^3^, total acquisition time (TA) = 10:49 min]. The images were used to identify the motor hand knob at the pre-central gyrus (Yousry et al., 1997) and to perform surface reconstructions of the cortical white–matter/gray–matter boundary.

Functional images were recorded using a gradient-echo EPI sequence at 1.5 × 1.5 × 1.5 mm^3^ resolution (TR = 2000 ms, TE = 36 ms, flip angle = 70°, acquisition matrix = 128 × 128, field of view = 192 × 192 mm^2^, partial Fourier factor = 6/8). Nineteen double-oblique (transverse-to-sagittal and transverse-to-coronal) sections were positioned perpendicular to the central sulcus, cutting the motor hand knob in mediolateral direction and covering its whole depth. The AutoAlign Scout feature (Siemens Healthcare, Erlangen, Germany) ensured an identical slice position for all consecutive fMRI runs for the individual subject.

A single gradient-echo EPI volume was performed with identical orientation and position as the functional measurements, but covering the entire brain with 81 sections (1.5 × 1.5 × 1.5 mm^3^ resolution, TR = 8600 ms, TE = 36 ms, flip angle = 70°, acquisition matrix = 128 × 128, field of view = 192 × 192 mm^2^, partial Fourier factor = 6/8), to facilitate registration of the partial-volume images to the whole-volume structural images. MR angiography was based on a T1-weighted 3D FLASH sequence (TR = 22 ms, TR = 4.43 ms, flip angle = 18°, resolution = 0.3 × 0.3 × 0.5 mm^3^, 57 sections from two overlapping slabs) with identical volume coverage and slice orientation as the functional images.

For three of the subjects, the second MRI session with all-phalanx mapping was repeated to assess the retest-reliability of the resulting maps by calculating the Euclidean distance between the peak-vertex coordinates of the two mapping sessions.

### Tactile stimulation and fMRI mapping

**Figure 1:**
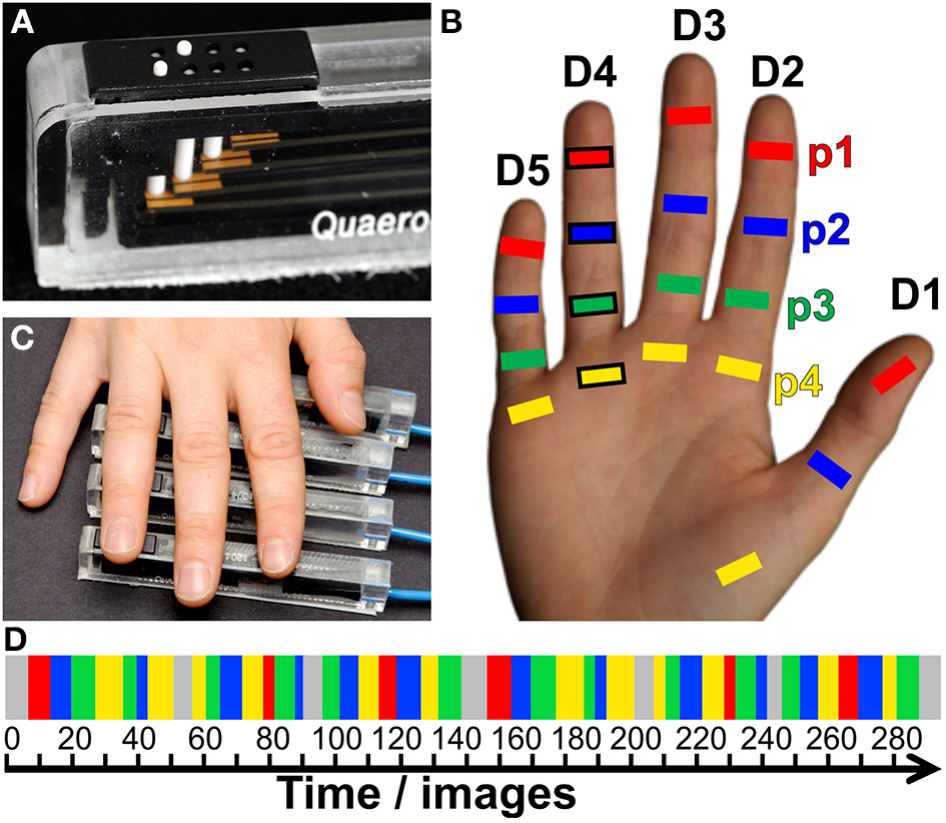
**Tactile stimulation. (A)** Piezo-electric device used for vibrotactile stimulation of individual phalanges. **(B)** Stimulated digit areas for mapping (p1, red; p2, blue; p3, green; and p4, yellow). **(C)** One digit was stimulated per run, the example refers to the positioning of the piezo-electric devices (black frames in **B**) to the ring finger. **(D)** Pseudo-randomized stimulation design (color code as above, baseline = gray).

Tactile stimulation was applied by a piezo-electric unit (QuaeroSys, St. Johann, Germany) controlling independent stimulation modules, each with an 8-dot Braille display (2 × 4 matrix, covering an area of 2.5 × 7.5 mm^2^) at its distal top face (Figure 1A) (Schweizer et al., 2008; Schweisfurth et al., 2011). Stimulation frequency was set to 32 Hz. In each cycle two out of the 8 pins were randomly chosen and elevated (square wave, stimulation duration = 10.4 ms, inter-stimulus interval = 20.8 ms) resulting in a fast varying stimulation pattern across the entire Braille display. The pins were set to maximum drive-out, to obtain a salient tactile stimulation which was clearly perceivable. Throughout the measurements subjects were told to keep their right hand relaxed and pronated.

For the digit localizer measurement, five stimulation modules were individually positioned under the distal phalanx (p1) of each digit of the right hand, with the Braille display being oriented centrally below the phalanx, parallel to the digit axis, with the bulk of the module pointing away from the digit. During the fMRI measurement sequential stimulation was applied, separately stimulating each fingertip from the thumb (D1) to the little finger (D5), with a rest period embedded after each fourth digit stimulation (D1, D2, D3, D4, Rest, D5, D1, D2, D3, Rest, D4, D5, D1, D2…). The duration of the stimulation at each site and for each rest period was 12 s (6 images). During the entire measurement each of the 5 digits was stimulated 8 times (total = 96 s, 48 images). Total measurement time including 10 distributed rest periods (total = 120 s, 60 images) and baseline (20 s, 10 images) added up to 10:20 min (310 images).

For intra-digit phalanx mapping tactile stimulation was applied to the first, second, and third phalanx (p1, p2, p3) and to the base (p4) of each digit (Figure 1B). The term “base” refers to the volar pads described in primate studies (Merzenich et al., 1978) and denotes the palmar skin position over the caput of the metacarpal bone proximal to the respective digit (Blankenburg et al., 2003). Each of the five fMRI measurements in the second MRI session explored the topography of the phalanges within a single digit. Four stimulation modules (only 3 for the D1 due to the missing p3) were individually positioned centrally below the phalanx, but orthogonally to the digit axis (Figure 1C). For stimulation of the phalanges of D2 and D3 the bulk of the modules pointed to the ulnar, for D4 and D5 to the radial, and for D1 to the ulnar (digit base and p2) and distal (p1) side. Each functional measurement (Figure 1D) consisted of 6 cycles of a stimulation block (average: 84 s, 42 images) alternating with a rest block (average: 12 s, 6 images). Within this stimulation block, the distal phalanx (p1) was stimulated once (12 s on average: jitter between 8, 12, or 16 s) and each of the other phalanges (p2, p3) and the base (p4) twice (2 × 12 s on average: jitter between 8, 12, or 16 s) within the block to account for the lower sensitivity of p2, p3, and p4 (Johansson and Vallbo, 1979; Schweisfurth et al., 2011). The succession of the four stimulation sites within the stimulation block was pseudo-randomized such that stimulation of one phalanx was always followed by stimulation of another phalanx or a rest block. During fMRI of one digit, the total stimulation time was 72 s (36 images) for the first phalanx (p1) and 144 s (72 images) for the phalanges p2, p3, and p4. Total scan time for the functional measurement, including baseline (20 s, 10 images), was 9:56 min or 298 images (7:32 min, 226 images for D1).

To assure attention on the stimulation, each functional measurement was associated with a task in which subjects were instructed to covertly count short randomly distributed interrupts embedded in the tactile stimulation (duration 156 ms, repetition every 0.5–3 s). After completion of the measurement, subjects reported the overall number of interrupts they counted and received verbal feedback from the experimenter about the actual number of interrupts during the measurement.

### Pre-processing and co-registration

The first part of data analysis was performed with BrainVoyager QX 2.3 (Goebel et al., 2006, Brain Innovation, Maastricht, The Netherlands). Structural T1-weighted images were corrected for intensity inhomogeneities, the axial slice orientation was brought parallel to the plane through the anterior and posterior commissures (AC-PC plane), and a cortical mesh at the white–matter gray–matter border was reconstructed. Functional runs were motion-corrected in k–space (Siemens Healthcare, Erlangen, Germany) and image space (BrainVoyager) as well as temporally high-pass filtered as implemented by default in BrainVoyager. In the same step, each functional run for each subject was registered to the first fMRI measurement (functional localizer) of the first session (6 degrees of freedom, using trilinear/sinc interpolation) to ensure an accurate alignment between all functional measurements within a subject. This first functional measurement of each subject was registered (6 degrees of freedom) to the individual whole-brain T1-weighted structural image; additional manual optimization ensured its best possible registration specifically in the region of the central sulcus. Using trilinear interpolation, the resulting registration matrix was then applied to each functional run of the subject to achieve identical projections onto the T1-weighted 3D data in AC-PC-oriented native space. During registration the fMRI data were also interpolated from 1.5 mm isotropic resolution to 1 mm isotropic resolution. No stereotactic transformation was applied in any of the performed registration steps.

The MR-angiography volume was evaluated within FSL (FMRIB Software Library, Smith et al., 2004), where it was registered to the T1-weighted image using FLIRT (FMRIB's Linear Image Registration Tool, Jenkinson and Smith, 2001). More precisely, the registration of the MR-angiography volume to the T1-weighted volume was achieved through concatenation of intermediate registration steps from the MR-angiography volume to the first volume of the functional localizer measurement to the whole-brain EPI volume to the T1-weighted volume. This resulted in transformation of the MR-angiography volume onto an interpolated 0.5 mm isotropic T1-weighted volume in BrainVoyager AC-PC-oriented native space.

The next step involved the exclusion of voxels around prominent blood vessels. This method is commonly applied in high-resolution fMRI studies of visual processing in humans (Cheng et al., 2001; Yacoub et al., 2001; Shmuel et al., 2007) to reduce the BOLD signal from these vessels, which limit the spatial specificity of the BOLD response (see Polimeni et al., 2010 for the visual system). For the somatosensory system this was based on a two-step approach. Using the functional localizer, fMRI activation clusters in which the peak vertices (of an activation cluster) of three or more digits were in very close proximity (not further than 3 mm apart in any direction) were identified, based on the assumption that such an extensive spatial coincidence of peak activation from the distal phalanx of three or more digits is not in line with the expected separate digit representations in BA 3b and could therefore represent a BOLD signal change due to a large blood vessel (Schweizer et al., 2010). In those cases the respective location in the high-resolution angiograms was inspected for the presence of a vessel. If a vessel could be observed at the respective position, the area activated (at q(FDR) ≤ 0.05) upon stimulation of the two assumingly most distally represented fingers (as expected from mediolateral fingertip somatotopy, e.g., D4 and D5 for positions close to the expected D1 representation or D1 and D2 for positions close to the expected representations for D4 and D5) was marked and respective vertices were excluded from further analyses.

### Data analysis

Stimulus-related activations were determined by a GLM analysis. For the functional localizer measurement, the main effect for the predictor of each of the 5 distal phalanges was calculated. For the intra-digit measurements, a GLM analysis for each digit was conducted calculating the main effects for the 4 predictors p1 to p4 (only p1, p2, and p4 for the thumb). All predictors were convolved with a two-gamma function (Friston et al., 1998) and voxels passing a positive *t*-value threshold corresponding to a false discovery rate of q(FDR) = 0.05 were considered significant. The resulting maps were projected onto the individual white–matter gray–matter surface reconstruction. The value at each vertex was calculated by taking into account the values of the statistical map voxels up to 3 mm orthogonal to the mesh, as it is standard in BrainVoyager 2.3. Further analysis of the resulting maps was restricted to BA 3b, here defined as being located at the posterior wall of the central sulcus. Because no *in-vivo* marker for the identification of BA 3b borders in individual subjects is available, somatosensory mapping studies use a schematic approach to define the putative BA 3b/1 border (suggested by Moore et al., 2000, used e.g., by Blankenburg et al., 2003; Nelson and Chen, 2008, and Stringer et al., 2011). Here, the junction of the superior portion of the posterior bank of the central sulcus with the beginning of the curve of the crest of the post-central gyrus was individually defined as the putative border between BA 3b and BA 1 (Moore et al., 2000). The medial-to-lateral extent of the putative BA 3b within this border was based on the extend of the individual p1 activations of the 5 digits measured in the first functional measurement, plus an addition of approximately 1 cm to either side of the most medial and lateral activation. After BA 3b definition, the mesh-spanning vertices of this area and their thresholded *t*-values within this BA 3b digit area were exported to MATLAB.

For further analysis, the coordinates of the mesh vertex with the highest *t*-value were determined for each phalanx of each finger. This peak-value approach is generally applied in somatosensory mapping studies (Kurth et al., 2000; Blankenburg et al., 2003; Nelson and Chen, 2008; Schweizer et al., 2008; Stringer et al., 2011). It does not take into consideration the description and comparison of the BOLD-activation overlap, which is beyond the scope of the present study.

To provide a distance measure for comparison of the present data with other studies, mean Euclidean distances were calculated for each phalanx within a set of homologous phalanges, between the phalanx' peak vertex and the respective thumb's phalanx peak vertex:

$$\sqrt{\begin{array}{c} {\left(x_{digit,phal.}-x_{D1,phal.}\right)}^{2}+{\left(y_{digit,phal.}-y_{D1,phal.}\right)}^{2}\\ +\;{\left(z_{digit,phal.}-z_{D1,phal.}\right)}^{2} \end{array}}$$

Analogously, for each phalanx within a digit, the mean intra-digit Euclidean distance between the phalanx peak vertex and the respective digit's first–phalanx peak vertex was calculated by

$$\sqrt{\begin{array}{c} {\left(x_{digit,phal.}-x_{digit,p1}\right)}^{2}+{\left(y_{digit,phal.}-y_{digit,p1}\right)}^{2}\\ +\;{\left(z_{digit,phal.}-z_{digit,p1}\right)}^{2} \end{array}}$$

To describe features of the map outline both within and across subjects, across-digit and intra-digit analyses were conducted, using the newly-developed two-step DiOr method as described below in detail for across-digit analysis.

**Figure 2:**
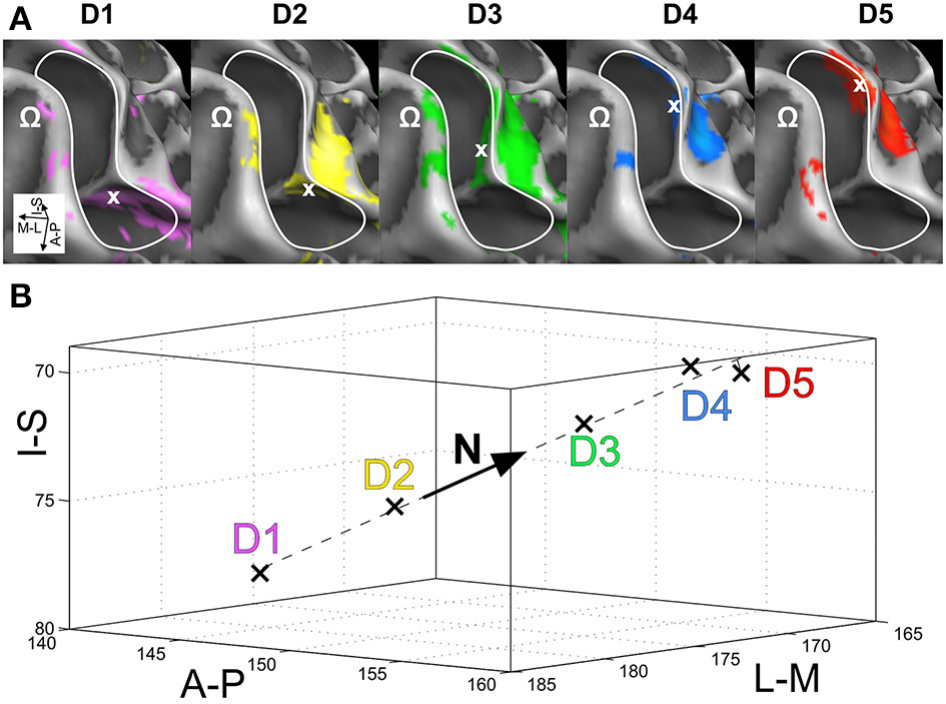
**Direction vector N (for p1 of subject S2). (A)** View from above onto the surface reconstruction of an individual left hemisphere, with the motor hand knob (Ω) in the foreground and the somatosensory BA 3b hand area in the center (A-P, anterior-posterior; M-L, medial-lateral; I-S, inferior-superior). The area used for peak–vertex analysis is marked (white border), peak vertices are marked by white crosses. From left to right, digit activations refer to D1, magenta; D2, yellow; D3, green; D4, blue; and D5, red. **(B)** Three-dimensional representation of the 5 peak vertices (1 mm isotropic coordinates in AC–PC-oriented native space) and their best-fitting line (dashed) as direction vector N. This subject shows somatotopy as the projections of the first phalanges onto N are sequential from D1 to D5.

In a first step of across-digit analysis, respective activation peaks were analyzed to determine the direction of largest variation for each set of homologous phalanges (p1 across 5 digits, p2 across 5 digits, p3 across 4 digits, p4 across 5 digits). A principle component analysis (PCA) was applied to the peak-vertex coordinates of the phalanx representations in order to extract their main direction of variation, i.e., the direction in AC-PC-oriented native space along which they were mostly distributed. For each subject and set of homologous phalanges the PC vector N = (Nx, Ny, Nz) was obtained (being the eigenvector belonging to the largest eigenvalue of the covariance matrix, normalized to a length of 1). It described the best linear fit and explained the largest amount of variance across the peak positions within a set of homologous phalanges (Figure 2). To ensure that the N vector pointed approximately into the direction of D1–D5, its sign was chosen such that the N coordinate with the largest absolute value had the same sign as the respective coordinate of the difference vector D5–D1 for across-digit analysis (i.e., if Nx>Ny and Nx>Nz, then if D5x–D1x>0, also Nx > 0). To investigate the possibility of a common direction pattern across subjects, for each set of homologous phalanges (and digit bases) the respective N coordinates along each of the three axes were compared across subjects to explore a possible across-digit direction consistency. To that end, first Fisher's transformation (take the *atanh* of the value, Fisher, 1915) was applied to the individual N vector coordinates and then one-sample *t*-tests (two-tailed, at *p* ≤ 0.017 corrected for testing along the three axes) against zero were calculated.

In addition to the direction along which the representations were aligned, we explored in a second step whether a D1-to-D5 succession could be detected along that direction. For each subject and set of homologous phalanges, it was assessed whether a D1-to-D5 ordering was observed across the 5 digits along the individual direction of largest variation. For that purpose, the respective peak vertices were transferred into an orthonormal coordinate system which included N as first base vector. After this transformation, the first coordinate by definition described the position of each phalanx representation along the line given by N. Using this information, it could be assessed whether the peak vertices were ordered (from D1 to D5) according to the definition that neighboring phalanges yield similar or sequentially increasing coordinates along the PC vector and that not all peak vertices are identical. Across subjects, binomial tests (*p* ≤ 0.05) then examined whether more subjects than expected by chance showed a D1-to-D5 succession for homologous phalanges (across-digit ordering). The DiOr approach used the probabilities to find such an ordered pattern in individual subjects (i.e., probabilities 0.056 for p1, p2, and p4 as well as 0.189 for p3, by assuming equal likelihoods for each possible pattern).

For the intra-digit analysis, we similarly assessed the individual directions along which the representations of a digit's phalanges were aligned and possibly ordered. To that end, we performed analogous steps as in the across-digit analysis but including the peak-vertex coordinates of the three phalanges within a digit (two phalanges for the thumb). In each intra-digit analysis, only those subjects were included that showed significant activation for all phalanges of the respective digit. For each subject and digit, the normalized PC vector N was calculated, defined such that it approximately pointed in the direction of p1–p3 (from p1 to p2 for the thumb). At group level, the N coordinates of a digit were compared to investigate a possible intra-digit direction consistency along any of the axes. For D2 to D5, a second analysis step assessed whether more subjects than expected by chance presented with ordered phalanges (from p1 to p3, intra-digit ordering) along the PC vector (i.e., probability 0.5 for D2 to D5). For D1, no such analysis was carried out, as there by definition always is a p1-to-p2 ordering.

## Results

In the present study, individual activation maps in BA 3b, covering each of the phalanges and digit bases of all right-hand digits, were obtained and analyzed. Common features such as a similar main direction of succession across subjects and ordering along this direction were described on two levels: for homologous phalanges (all p1, all p2, all p3, or all p4) across digits and for different phalanges within single digits.

To control for attention on the tactile stimulation, subjects had to covertly count short randomly-distributed interrupts of the tactile stimulation. Because there was on average only a 4% difference between the actual and reported numbers for each finger across subjects, it can be assumed that subjects were able to focus their attention toward the stimulation.

Activation patterns acquired during the functional localizer measurements, stimulating the most distal phalanges (p1) of each of the five digits, generally showed the expected medial-to-lateral succession of digit representations from D5 to D1 along the posterior wall of the central sulcus.

After the first session, 3 of the 18 subjects had to be excluded, either due to no detectable activation in the assumed BA 3b, or dominance of a large vessel parallel to the entire SI digit area, or a rare variation of a divided central sulcus at the level of the hand knob (Alkadhi and Kollias, 2004).

Out of the remaining 15 subjects, in a second session complete tactile stimulation of all 4 within-digit stimulation sites could be accomplished in all subjects for D5, in 14 subjects for D4, D3, and D2, and in 12 subjects for D1 (all 3 sites). Missing measurements resulted from difficulties in positioning the piezo-electric modules or from fatigue of the subjects due to the lengthy session. After statistical analysis, projection to the surface reconstruction, and elimination of assumed large vessel contribution, significant BA 3b activations of all phalanges and the base within a digit were present in 11 subjects for D5, D4, and D1, in 12 subjects for D3 and in 14 subjects for D2. The complete map, i.e. detectable activation from all phalanges and all bases of all digits (total of 19 stimulation sites) was available in 7 subjects.

Averaging across the 19 stimulation sites in these seven subjects, a significant activation consisted of 62.95 ± 86.51 voxels [mean ± standard deviation (SD)], and the average peak-voxel's t-value was 5.50 ± 1.59 (mean ± SD). Numbers of voxels and *t*-values per phalanx and digit are presented in Table 2.

**Table 2:** **Volume and *t*-values of BOLD activations across and within digits**.

**Volume: mean ± standard error of the mean (SEM) (median) [mm^3^]**				***t*-value: mean ± SEM (median) [mm^3^]**			
**Average across digit**		**Average across phalanx**		**Average across digit**		**Average across phalanx**	
D1	48 ± 2 (25)	p1	82 ± 1 (59)	D1	5.49 ± 0.05 (5.36)	p1	6.25 ± 0.03 (5.99)
D2	58 ± 1 (32)	p2	69 ± 2 (47)	D2	5.26 ± 0.03 (5.06)	p2	5.57 ± 0.03 (5.32)
D3	62 ± 2 (40)	p3	47± 1 (24)	D3	5.67 ± 0.03 (5.30)	p3	5.27 ± 0.02 (5.06)
D4	92 ± 3 (40)	p4	52 ± 2 (25)	D4	5.68 ± 0.04 (5.30)	p4	4.76 ± 0.03 (4.75)
D5	53 ± 1 (42)			D5	5.45 ± 0.03 (5.17)		

*The first and third column present the average volume and t-values across all phalanges for each digit. The second and fourth column depict the average volume and t-values across all homologous phalanges*.

**Figure 3:**
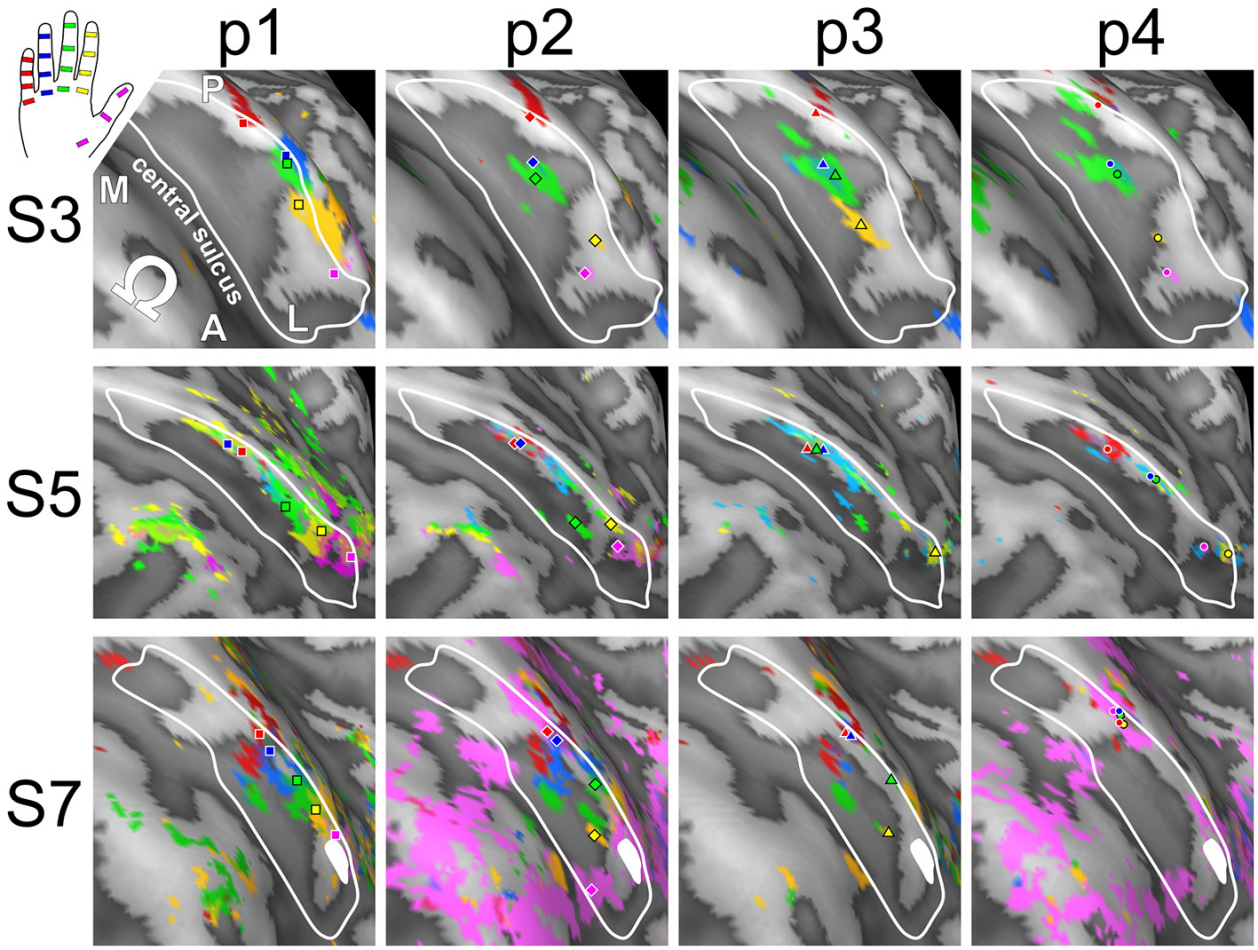
**Across-digit representations of phalanges and bases (subjects S3, S5, and S7)**. View from above onto the inflated surface reconstruction of individual left hemispheres with the motor hand knob (Ω) in the foreground and the somatosensory BA 3b hand area in the center (convex areas, light gray; concave areas, dark gray; putative vessels, white; A-P, anterior-posterior; M-L, medial-lateral). The area used for peak–vertex analysis is marked (white border). Each column shows the activations for homologous phalanges p1, p2, and p3 as well as digit bases p4. Peak voxels are illustrated in the respective color. D1, magenta; D2, yellow; D3, green; D4, blue, and D5, red.

**Figure 4:**
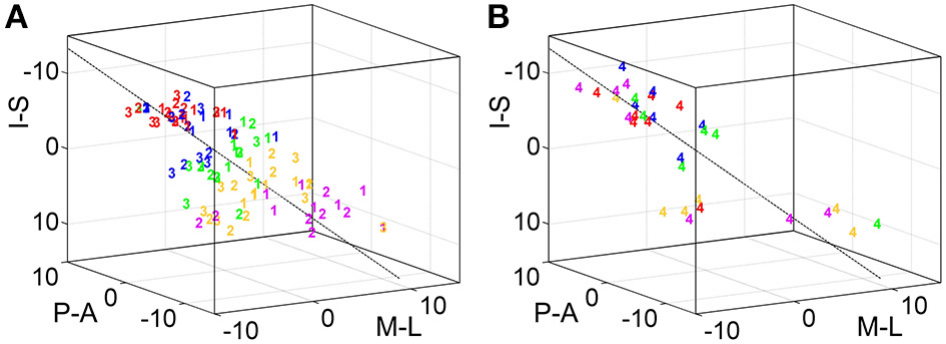
**Organization of digit area in BA 3b**. For each of the 7 subjects that presented with significant activation for all phalanges and bases, all peak vertices were normalized by the respective subject's mean value across phalanges (but not bases; A-P, anterior-posterior; M-L, medial-lateral, I-S, inferior-superior; D1, magenta; D2, yellow; D3, green; D4, blue; D5, red; all units in 1 mm). **(A)** Normalized peak-vertex coordinates for p1 (=1), p2 (=2), and p3 (=3). **(B)** Normalized peak-vertex coordinates for p4 (=4).

Visual inspection on the surface reconstruction confirmed the known succession of digit activations for the first phalanx (p1), progressing from medial-posterior-superior (D5) to lateral-anterior-inferior (D1) (Figure 3, first column). The data from the additional stimulation sites revealed a similar pattern for the second and third phalanx, as both sets of homologous phalanges (all p2 and all p3) exhibited the analog succession from medial-posterior-superior (D5) to lateral-anterior-inferior (D1) (Figure 3, second and third column). This succession is also evident in the peak-voxel distribution, as illustrated in Figure 4A, where the peak vertices of all stimulation sites are plotted in 3D coordinate space. The figure also shows that the peak phalanx activations of the same digit tend to group together, resulting in an overall D5-to-D1 succession of the phalanx representations across the five digits. The finger-base (p4) representations, in contrast, showed a much more variable pattern across digits and subjects (Figure 4B), ranging from the vicinity of (or even overlap with) the respective phalanx activations (Figure 3, subject S3) to positions lateral to D1 (rarely) or more commonly medial to D5 (Figure 3, subject S7).

This visually-observed succession for each of the three phalanges and variability of the finger-base representations were reflected in the Euclidean distances between the peak vertices of homologous-phalanx representations (Table 3). The distance between D1 and D5 was 16.7 ± 4.2 mm for p1, 14.7 ± 7.0 mm for p2, 18.0 ± 3.2 mm for p3, and 11.2 ± 8.4 mm for p4. These rather similar D1–D5 distances for p1, p2, and p3 resulted from the strong overlap of the BOLD activations and the relatively closely located peak vertices of p1, p2, and p3 (and sometimes p4) within the digits. Compared to the D1–D5 distances for p1, p2, and p3, the value for the digit base (p4) tended to be smaller and more variable, pointing to the visually-observed variability of the p4 peaks for the digits D3, D2, and D1.

**Table 3:** **Euclidean distance across digits and within digits**.

**Distance to D1/mm**	**D2**	**D3**	**D4**	**D5**
p1 (to p1 of D1)	6.8 ± 2.1	11.4 ± 3.2	15.1 ± 5.7	16.7 ± 4.2
p2 (to p2 of D1)	4.6 ± 3.0	9.2 ± 5.1	13.3 ± 7.0	14.7 ± 7.0
p3 (to p2 of D1)	8.1 ± 2.3	12.8 ± 3.8	15.9 ± 4.1	18.0 ± 3.2
p4 (to p4 of D1)	6.9 ± 7.6	12.6 ± 9.7	8.2 ± 7.0	11.2 ± 8.4
**Distance to p1/mm**	**p2**	**p3**	**p4**	
D1 (to p1 of D1)	4.8 ± 2.6		14.4 ± 8.7	
D2 (to p1 of D2)	4.0 ± 2.8	6.2 ± 2.1	9.2 ± 4.2	
D3 (to p1 of D3)	2.9 ± 2.7	6.6 ± 5.2	8.8 ± 5.6	
D4 (to p1 of D4)	2.9 ± 2.9	5.0 ± 3.1	6.8 ± 3.0	
D5 (to p1 of D5)	2.3 ± 2.4	3.4 ± 2.3	5.4 ± 5.0	

*For each phalanx within a set of homologous phalanges (upper part of table), the across-digit Euclidean distance (mean ± standard deviation) between the phalanx peak voxel and the respective thumb's phalanx peak voxel are stated (including the 7 subjects showing activation for each finger and phalanx). For each phalanx within a digit (lower part of table), the intra-digit Euclidean distance (mean ± standard deviation) between the phalanx peak voxel and the respective digit's first–phalanx peak voxel are stated (same subjects as above)*.

The within-digit Euclidean distance between the proximal (p3) and the distal phalanx (p1) (Table 3) exhibited comparable values for the digits D1 through D4: 4.8 ± 2.6 mm for D1, 6.2 ± 2.1 mm for D2, 6.6 ± 5.2 mm for D3, 5.0 ± 3.1 mm for D4, and a reduced value of 3.4 ± 2.3 mm for D5. Here, standard deviations across measurements were comparable, pointing to a smaller p1–p3 distance for D5. The Euclidean p1–p4 distance increased across the digits from D5 to D1 (Table 3), which has to be at least partly ascribed to the much higher spatial variability of the p4 representations of D1, D2, and D3 being distributed across the whole digit area.

**Figure 5:**
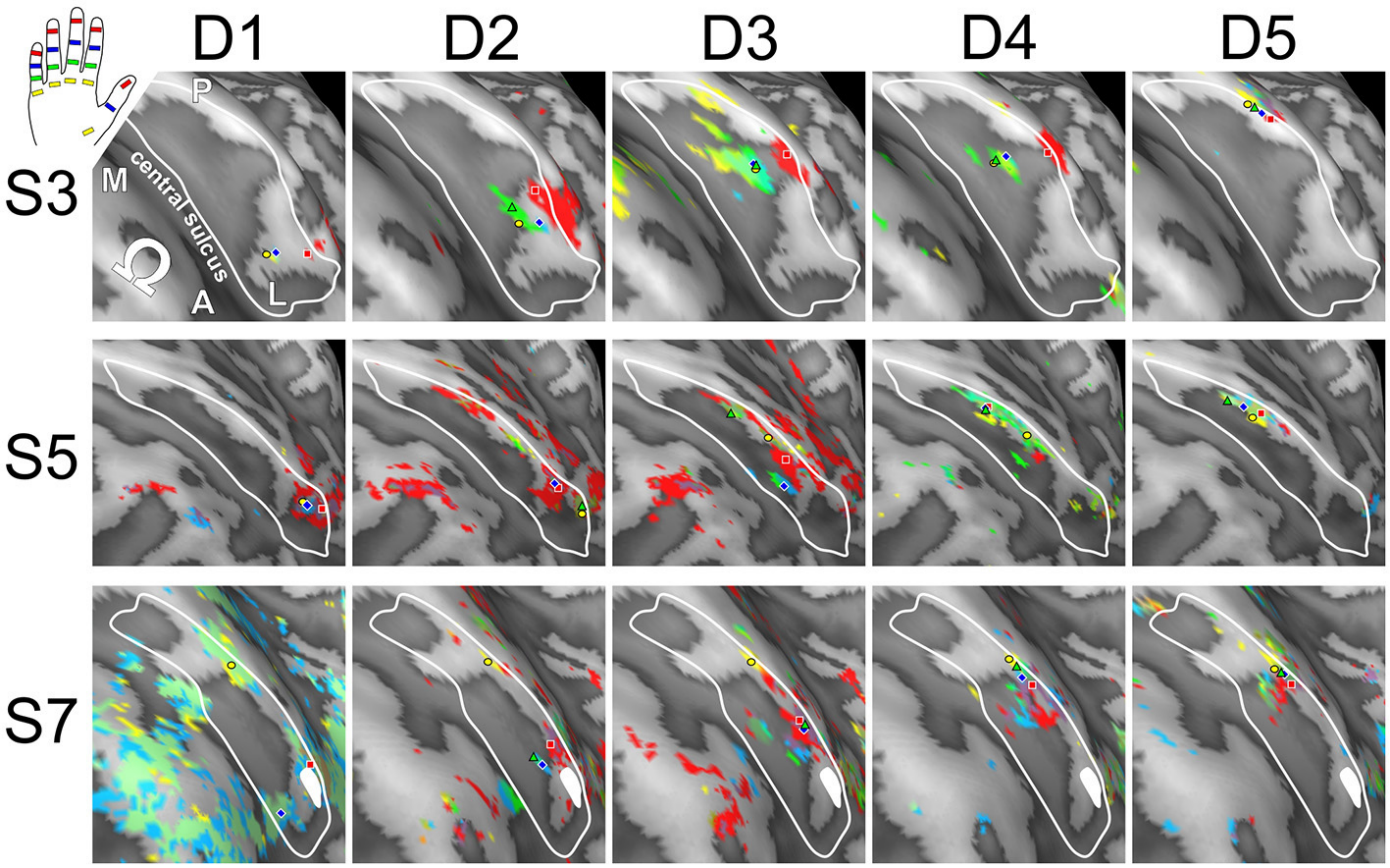
**Intra-digit representations of phalanges and bases (subjects S3, S5, and S7)**. View from above onto the inflated surface reconstruction of the left hemisphere with the motor hand knob (Ω) in the foreground and the somatosensory BA 3b hand area in the center (convex areas, light gray; concave areas, dark gray; putative vessels, white; A-P, anterior-posterior; M-L, medial-lateral). The area used for peak–vertex analysis is marked (white border). Each column shows the activations for phalanges p1 (= red), p2 (= blue), and p3 (= green) as well as base p4 (= yellow) of a single digit (D1 to D5), peak voxels are illustrated in the respective color.

Visual inspection of the within-digit maps (Figure 5) indicated that subjects generally showed a similar p1-to-p3 (or even p1-to-p4) succession within the expected digit area for D5 and to a lower degree for D4. All other within-digit maps visually presented with a high level of individuality, both at the volume and at the surface level.

The retest reliability across sessions (evaluated in 3 subjects) revealed a mean Euclidean difference between the peak vertices of the two measurements of 2.9 ± 3.6 mm (averaged across the 3 subjects and stimulation sites).

In the following two subsections, we report the results of the statistical evaluation of the across- and intra-digit maps.

### Statistical evaluation of across-digit maps with DiOr

**Figure 6:**
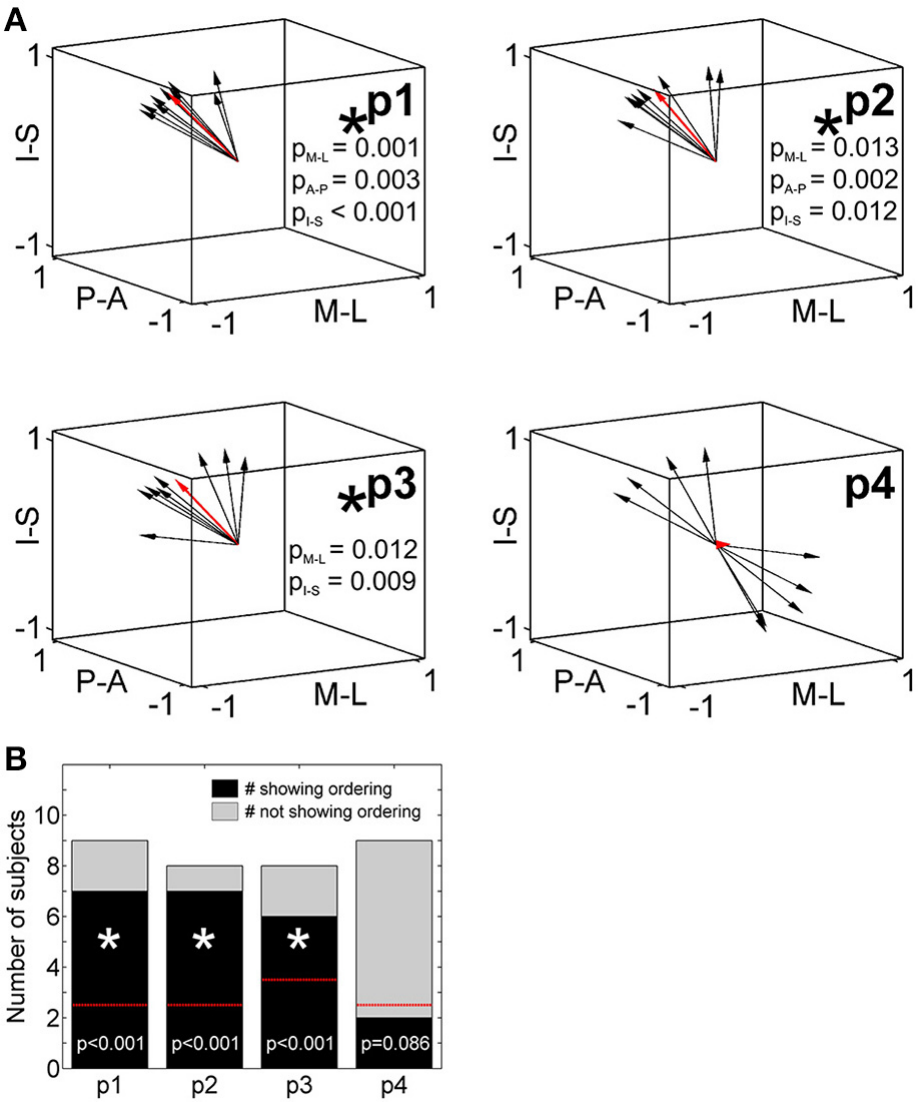
**Across-digit direction consistency and ordering. (A)** Across-digit direction vectors for p1 to p4 (black arrows, individual direction vectors; red arrows, mean values averaged across subjects; A-P, anterior-posterior; M-L, medial-lateral; I-S, inferior-superior). Across-digit direction consistency is indicated by an asterisk and respective *p*-values. **(B)** Across-digit ordering as given by the number of subjects showing (black) or not showing (gray) ordered activations from D1 to D5 along the individual N vectors. The red lines illustrate the minimal number of ordered subjects needed to observe across-digit ordering. Phalanges showing ordering are indicated by an asterisk and respective *p*-values.

To statistically compare across-digit representation patterns across subjects, we determined the main spatial directions along which the homologous phalanges and the digit bases were aligned across the five digits (Table 4). For the distal, second, and third phalanges (p1, p2, and p3) individual direction vectors were similarly oriented across subjects, as averaging these individual, normalized vectors across subjects resulted in a mean direction vector of substantial and comparable length for the three phalanges (see red vector in Figure 6A). Statistically, across-subjects *t*-tests (against zero) revealed significant across-digit direction consistency along all three axes, not only for p1 but also for p2 and p3 (for p3 along the AP axis only in a trend; Table 4). The average direction vectors for the three phalanges pointed from lateral-anterior-inferior to medial-posterior-superior. For the digit bases (p4), such an across-digit direction consistency could not be observed, as the direction vectors of different subjects pointed to different directions, resulting in a small average direction vector across subjects (Figure 6A, Table 4).

**Table 4:** **Across-digit direction consistency**.

**Phalanx**	**p1**			**p2**			**p3**			**p4**		
	**M-L**	**A-P**	**I-S**	**M-L**	**A-P**	**I-S**	**M-L**	**A-P**	**I-S**	**M-L**	**A-P**	**I-S**
Mean N	−0.39	0.47	0.68	−0.28	0.52	0.70	−0.34	0.43	0.65	0.11	−0.06	0.01
SD	±0.23	±0.34	±0.14	±0.24	±0.28	±0.22	±0.27	±0.36	±0.35	±0.51	±0.54	±0.74
*p*−value	0.001^*^	0.003^*^	<0.001^*^	0.013^*^	0.002^*^	0.012^*^	0.012^*^	0.026	0.009^*^	0.520	0.770	0.466

*Mean N vectors (calculated across D1-to-D5 peak vertices), respective coordinates and standard deviations (SD) as well as p-values for phalanges p1 to p3 and digit bases p4 along the medial-lateral (M-L), anterior-posterior (A-P), and inferior-superior (I-S) ACPC axis, respectively*.

* *p ≤ 0.0167 (i.e., p ≤ 0.05 corrected for the three comparisons along the three axes)*.

To determine a possible ordering between homologous phalanges or digit bases along the direction vector, it was tested whether the projections of the respective peak vertices onto their best-fitting line (i.e., the direction vector N) were ordered from D1 to D5. For p1, p2, and p3, most subjects displayed this expected D1-to-D5 ordering which could statistically be confirmed not only for the first but also for the second and third phalanges (Figure 6B). In the 6 out of 25 individual cases in which the expected across-digit ordering was not observed, the swap consistently occurred between neighboring digits: three times between D4/D5 (see e.g., Figure 3, p1 in Subject S5), twice between D3/D4 (see e.g., Figure 3, p3 in Subject S5), and once between D1/D2. For the digit base, on the contrary, no across-digit ordering could be observed, as only 2 out of 9 subjects showed D1-to-D5 ordering.

Putting together the results from the direction-consistency analysis and the ordering analysis, for p1, p2, and p3 we generally found the D1-to-D5 representations to be ordered along a similar axis across subjects, pointing to an across-digit somatotopy for each of these phalanges.

### Statistical evaluation of intra-digit maps with DiOr

**Figure 7:**
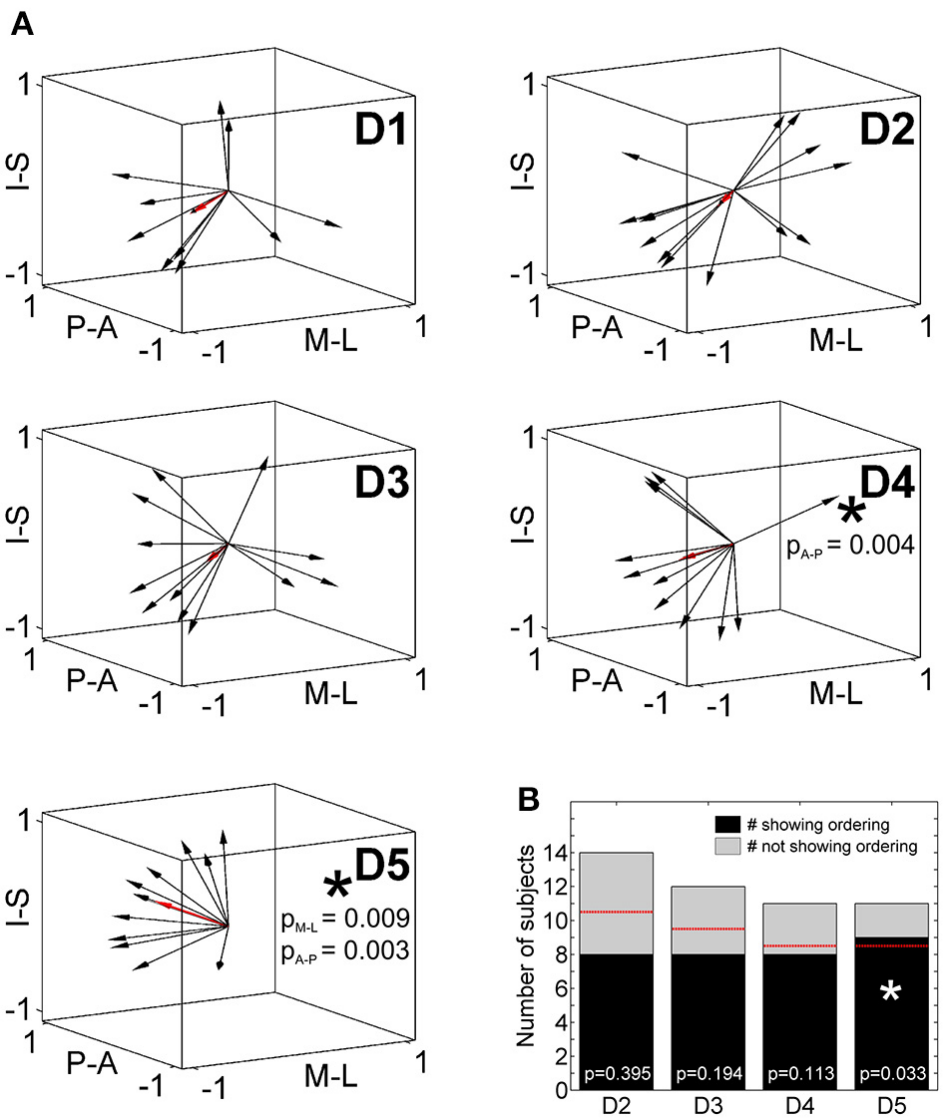
**Intra-digit direction consistency and ordering. (A)** Intra-digit direction vectors for D1 to D5 (black arrows, individual direction vectors; red arrows, mean values averaged across subjects; A-P, anterior-posterior; M-L, medial-lateral; I-S, inferior-superior). Intra-digit direction consistency is indicated by an asterisk and respective *p*-values. **(B)** Intra-digit ordering as given by the number of subjects showing (black) or not showing (gray) ordered activations from p1 to p3 along the individual N vectors. The red lines illustrate the minimal number of ordered subjects needed to observe intra-digit ordering. Digits showing ordering are indicated by an asterisk and respective *p*-values.

To statistically compare intra-digit representation patterns across subjects, for each subject and digit the main intra-digit direction was determined (Figure 7A), i.e., the principal direction along which the phalanges within a single digit were aligned in a subject. Only for the digits D4 and D5, similar directions were found across subjects (Table 5, Figure 7A). D5 exhibited a significant intra-digit direction consistency from lateral-anterior to medial-posterior. For D4, an anterior-to-posterior intra-digit direction consistency was observed. The direction vectors of D1, D2, and D3 did not show any consistency across subjects.

**Table 5:** **Intra-digit direction consistency**.

**Digit**	**D1**			**D2**			**D3**			**D4**			**D5**		
	**M-L**	**A-P**	**I-S**	**M-L**	**A-P**	**I-S**	**M-L**	**A-P**	**I-S**	**M-L**	**A-P**	**I-S**	**M-L**	**A-P**	**I-S**
Mean N	−0.27	0.16	−0.25	−0.12	0.06	−0.17	−0.10	0.21	−0.24	−0.30	0.37	−0.22	−0.43	0.46	0.23
SD	±0.59	±0.40	±0.64	±0.56	±0.55	±0.64	±0.50	±0.58	±0.62	±0.46	±0.33	±0.69	±0.39	±0.34	±0.58
*p*−value	0.137	0.238	0.280	0.526	0.940	0.360	0.822	0.449	0.303	0.134	0.004^*^	0.192	0.009^*^	0.003^*^	0.162

*Mean N vectors (calculated across the p1 to p3 peak vertices), respective coordinates and standard deviations (SD), and p-values for digits D1 to D5 along the medial-lateral (M-L), anterior-posterior (A-P), and inferior-superior (I-S) ACPC axis, respectively*.

* *p = 0.0167 (i.e., p ≤ 0.05 corrected for the three comparisons along the three axes)*.

To determine the intra-digit ordering for each digit (except for D1), it was tested whether the projections of the peak vertices onto their direction vector were ordered from p1 to p3. Only for D5 (but for no other digits) more subjects (9 out of 11) than expected by chance exhibited an ordered phalanx representation (Figure 7B). For D4, significance was missed by only a single subject, while no intra-digit ordering could be observed for D3 and D2.

Putting together the results from the direction-consistency analysis and the ordering analysis, for D5 we generally found the p1-to-p3 representations to be ordered along a similar axis across subjects, pointing to an intra-digit somatotopy for the little finger. A similar trend was observed for the ring finger, while D1, D2, and D3 presented with highly individual intra-digit maps.

As additional test, p1 to p3 of D3 and D5 were measured in six different subjects with a three-fold higher stimulation time per phalanx (unpublished data). For D5, the results showed a similar N-vector as in the present study yielding p1-to-p3 ordering in 5 out of 6 subjects. For D3, despite the increased amount of measurement time, again in line with the current work only 3 subjects presented with ordered p1-to-p3 representations.

## Discussion

BOLD MRI activation peaks, elicited by tactile stimulation of the phalanges and digit bases of all digits, were analyzed to quantitatively describe the somatotopic maps in BA 3b within and across individual subjects. Due to the complexity of the data, they were not only described by standard measures as Euclidean distances, but also evaluated by the suggested DiOr approach, looking at the main direction of alignment and a possible ordering along that direction. Across-digit analysis considering homologous phalanges across the 5 digits showed the D1-to-D5 succession of the distal, medial, and proximal phalanges along the central sulcus. The digit bases presented with a partially divergent cortical pattern. Intra-digit analysis revealed increasing Euclidean distances along the phalanges, but only D5 and D4 presented with a consistent direction of alignment and only D5 with an ordering of p1, p2, p3 along this direction. Hence, only for D5 (and in a trend: D4) intra-digit maps resembled each other between subjects.

### Comprehensive functional maps of all phalanges and digit bases

The challenge to map the entire digit area of the right hand was met by combining an optimized stimulus paradigm including an attentional task with high-resolution fMRI: The attempt allowed for measuring all phalanges and digit bases in one session. Under these conditions, significant activations could be obtained for most stimulation sites in 15 subjects, out of which 7 subjects showed activation for all 19 stimulation sites. The sizes of the elicited activations were in the expected range. The similar statistical *t*-values of the activations at the different stimulation sites approved our approach of stimulating the medial and distal phalanges as well as the digit base twice as often as the distal phalanges, thereby compensating for the weaker fMRI signal due to the smaller cortical representations of the former. The exemplary re-test measurements demonstrated comparable maps across sessions, with a distance of less than 3 mm (<2 voxels) between respective peak vertices of the two sessions.

### Across-digit findings

The quantitative activation maps of the homologous phalanges and digit bases across digits in BA 3b showed the succession of the phalanges from D5 to D1 along the general course of the central sulcus (from medial to lateral, superior to inferior, and posterior to anterior).

The increase of the average Euclidean distances between the peak representations of the distal phalanges of D2, D3, D4, and D5 and the one of D1 is generally interpreted as a reliable indicator of the expected succession of the individual digit representations from D5 to D1 along the central sulcus (e.g., Nelson and Chen, 2008) and is in complete concordance with previous fMRI studies describing the layout of the human digit area in BA 3b (Maldjian et al., 1999; Kurth et al., 2000; Nelson and Chen, 2008; Schweizer et al., 2008; Sanchez-Panchuelo et al., 2010). However, this interpretation can be problematic, as the D1-to-D5 representations could still be distributed in an unordered way even if there is a succession in the distances. To conclude from increasing Euclidean distances that the representations are successive in space, all D2-to-D5 representations must in addition be found in the same direction of the D1 representation. This assumption is not required for the DiOr approach applied here, the results of which are discussed in the next paragraph.

For the distal phalanx, the individual direction vectors (representing the main direction of alignment of the five activation peaks in each subject) all pointed into a similar direction. Further on, most of the subjects showed an ordered p1-representation succession from D5, D4, D3, D2 to D1 along this direction. These results demonstrate that the proposed DiOr analysis provides a valid and sensitive strategy to capture and statistically compare the layout of p1 maps from individual subjects as well as to extract general features as the well-known medial-to-lateral p1 somatotopy.

The activations elicited by stimulation of the medial and proximal phalanges revealed an analogous pattern of increasing distances from D1 toward D2, D3, D4, and D5, hinting to an equivalent representation succession for the medial as well as for the proximal phalanges from D5 to D1. This was further corroborated by the DiOr analysis, where both the medial as well as the proximal phalanges exhibited very similar across-digit directions across subjects, again pointing along the central sulcus. Also, the representations were generally ordered along these directions, showing the corresponding succession from D5 to D1 for the medial phalanges and from D5 to D2 for the proximal phalanges. Consequently, not only the distal phalanges, so far used as representatives of the entire digits, showed this succession, but also the lower phalanges of the digits. Along with visual inspection, this finding indicates that the representations of all phalanges of a single digit in BA 3b are located in relatively close vicinity, thereby forming a distinct area for each digit with relatively little overlap with the activations of other digits' phalanges. As these observations are analogous to the digit organization seen in electrophysiological mapping studies of squirrel, owl, and macaque monkeys (Merzenich et al., 1978; Sur et al., 1982; Iwamura et al., 1983), this succession can be described as the axis of digit representation along the course of the central sulcus, in analogy to the eccentricity axis along the calcarine sulcus of the primary visual cortex (Sereno et al., 1994). We hence propose a well-ordered succession of the D1-to-D5 representations as single digit entities along the central sulcus, where each single digit representation area is composed of the representations of the three phalanges. Looking at individual subjects, Sanchez-Panchuelo et al. (2014) found such ordered digit entities for D2 to D4 by simultaneous stimulation of p1, p2, and p3. Our results are in line with and extend such data, as we could demonstrate a succession separately for each phalanx.

For the digit bases (p4), we could not detect a consistent pattern of cortical representations across subjects. The Euclidean distance between the D1 representation and the D2, D3, D4, and D5 representations of the bases did not steadily increase such as in the phalanges. Digit–base peak representations were in some cases located close to the activations of the respective digit's phalanges, but in other cases medial to the digit area of D5 or lateral to that of D1. This variability was reflected both in the absence of a D1-to-D5 ordering of the digit bases in most of the subjects and in the large between-subjects variation of the within-base direction vectors. Our observations resembled a mixture of several previous findings concerning the location of the digit-base representation. Penfield and Rasmussen (1950) distinguished a separate hand area, located medial to D5, from the representational areas for each finger. In owl-monkey maps, the digit-base representations were described posterior to the p1-to-p3 representations (Merzenich et al., 1978). In macaques, the digit-base representations were found at both ends of the digit representations: medial-posterior to the little finger for D5 and D4 and lateral-posterior to the thumb for D3 and D2 (Kaas et al., 1979; Nelson et al., 1980; Iwamura et al., 1983).

### Intra-digit findings

The intra-digit analysis showed a much lower degree of consistency across subjects than the across-digit analysis. For each digit, the average Euclidean distance to the p1 representation increased from the medial to the proximal phalanx to the digit base. This might generally be interpreted as hint for a succession of the phalanx representations across the single-digit area. However, as discussed above, this only holds true if all lower phalanges are represented toward the same direction from the respective p1. As discussed in the next paragraph, this was only the case for some digits.

Analyzing the individual directions along which the intra-digit representations were aligned and potentially ordered revealed a more differentiated view of the layout of the maps within the single digits. Across subjects, only the digits D5 and D4 presented with a similar axis along which phalanx activations were distributed and only D5 exhibited an ordering from p1 to p3 (for D4, significance was missed by one subject).

In Table 1, all previous studies on intra-digit mapping in individual humans have been summarized. In the following paragraphs, the current findings are discussed in the light of this literature.

For D5, our current observations confirm and extend the results of our previous study (Schweisfurth et al., 2011), demonstrating a consistent arrangement between p1 and p4 for D5 but not for D2 across subjects. The common direction along which the D5 representations were ordered pointed from lateral (p1) to medial (p4) in the previous and lateral-anterior (p1) to medial-posterior (p3) in the present study. Both results indicate an intra-digit succession along the general course of the central sulcus, not perpendicular to it as in anesthetized owl and macaque monkeys (Merzenich et al., 1978; Nelson et al., 1980). One reason for this finding may be the relatively small extent of the D5 digit area. This small D5 area is suggested by the observed Euclidean p1–p3 distances, which showed a tendency to decrease from D1 toward D5, a trend in accordance with the maps of awake macaques (Iwamura et al., 1983). The claim is also corroborated by inspection of other mapping studies, where the volume of the p1 representation in BA 3b seems to decrease from D1 to D5 and to get closer to the one of BA 1 (Kurth et al., 2000; Nelson and Chen, 2008). The smaller anatomical D5-area extent along with the small distance between the BA 3b D5 representation and the putative cytoarchitectonical BA 3b/1 border might explain the shift of the p2 and p3 representations into the medial direction.

For D3, D2, and D1, the directions along which the phalanges were arranged differed substantially between subjects. In addition, no intra-digit ordering between phalanges along the individual direction vectors was found for D3 or D2. For D2, these results are in agreement with our previous study, where no consistent arrangement was observed between tip and base (Schweisfurth et al., 2011). However, this is in contrast to studies in anesthetized monkeys (Merzenich et al., 1978; Nelson et al., 1980) and two single-digit fMRI studies (Blankenburg et al., 2003; Sanchez-Panchuelo et al., 2012). Blankenburg and colleagues reported a rostral(p1)-to-caudal(p4) succession of phalanx representations of D3 across BA 3b despite coarser spatial resolution and as a result of a group analysis of 8 subjects. Factors contributing to the divergence between their result and the present work may be our increased spatial resolution, our higher number of subjects, and our analysis of individual maps. In the study of Sanchez-Panchuelo et al. (2012), the authors identified map reversals of the p1-to-p3 representations of D2 in SI, although no objective measure was given to justify the identification of the mirror-reversal positions. The across-subjects variation in the direction of the mirror reversal of the maps as well as the presence of ordered maps in only 4 out of 6 subjects seem to be comparable to the variations in direction and ordering of our intra-digit representations of D2 (ordered maps observed in 8 out of 14 subjects). The divergent results for anesthetized monkeys also have to be put into perspective by the awake-macaque study (Iwamura et al., 1983) reporting that the narrow zone of proximal-phalanx (p2/p3) representations located toward the BA 3b/1 border additionally contained receptive fields of distal phalanges. This kind of intermittent spread of p1 representations into the proximal-phalanx representation area could explain the divergent ordering and directions of phalanx representations within D3 and D2 among our subjects.

Finally, the question arises whether methodological issues could be responsible for not observing consistent within-digit somatotopies across subjects in some digits. The presence of a consistent within-digit somatotopy in D5, exhibiting the smallest digit representational area of all digits, is a clear indicator that the data and the applied DiOr analysis were adequate to detect somatotopic ordering across subjects. One limitation to be considered is the linearity of our DiOr approach, an anatomical simplification which does not take into account the complex anatomy of individual central sulci. We therefore carried out careful visual inspections of the D1, D2, and D3 maps which confirmed the results of our objective analysis procedure and assured that no existing somatotopy was missed due to the linear simplification. The approach of measuring the complete map of all phalanges in one session (in order to omit the influence of across-session variations) came with the price of a decreased number of averages per stimulation site, which in turn results in a slightly lower across-session reliability compared to our previous study (Schweisfurth et al., 2011). However, as results of additional measurements for D3 and D5 (unpublished data, see Results Section) with three-fold higher stimulation time are in line with the present results, the lower number of averages should not have been responsible for not observing somatotopy in some digits.

The reported inter-individual map variations within some but not all digits correspond to the general context of individual variability of representational maps in the somatosensory cortex. Individual differences in digit maps could be explained by the individual usage of the fingers, shaping the cortical representational maps in a usage-dependent manner, as suggested and shown in monkeys (Merzenich et al., 1987; Jenkins et al., 1990; Recanzone et al., 1992) as well as in humans (Elbert et al., 1995; Sterr et al., 1998; Braun et al., 2000). The findings reported here might have resulted from a rather uniform use of the mainly “supporting” digits D4 and D5, contrasted by the individually varying use of the digits D1 to D3 of the dominant hand, which might have led to more individualized cortical intra-digit maps for these digits.

## Conclusions

The present fMRI study describes the comprehensive map of all phalanges and digit bases across all digits of the dominant hand. An in-depth exploration of individual digit somatotopy was performed both across and within all five digits in a larger number of subjects. The novel quantitative approach DiOr described the individual directions along which the BA 3b BOLD-activation peaks aligned as well as the ordering along these directions.

Separate analysis of the representations of the medial and proximal phalanges across the five digits revealed an ordered succession from medial (little finger) to lateral (thumb) along the central sulcus, extending the well-known D1-to-D5 succession of the distal-phalanx representations. Hence, we propose a well-ordered succession of the representations of the five digits as entities along the central sulcus, each entity comprised of the representations of the three phalanges.

Within single digits, ordered phalanx representations (from p1 to p3) along a similar direction (across subjects) could be shown for D5 and in a trend for D4. For the digits D3, D2, and D1, the phalanx representations were neither generally ordered from p1 to p3 nor were they aligned along a similar direction across subjects, showing that the detailed arrangement of the phalanx representations within the intra-digit map varied between subjects. The lower degree of consistent intra-digit somatotopy for these digits might be related to a higher degree of usage-dependent plasticity.

This study underlines, based on high-resolution fMRI studies of the entire digit area in human somatosensory cortex, the importance of quantitative across-digit and within-digit maps, to not only extract general organizational features, but also to adequately deal with the complexity of individual map structures.

### Conflict of interest statement

The authors declare that the research was conducted in the absence of any commercial or financial relationships that could be construed as a potential conflict of interest.
